# P01-16 EUMOVE Project: an Erasmus+ Project for the promotion of healthy lifestyles among children and adolescents

**DOI:** 10.1093/eurpub/ckac095.016

**Published:** 2022-08-29

**Authors:** Sánchez-Oliva David, García-Calvo Tomás, Sánchez-López Sánchez-López, José Castro-Piñero, Alberto Grao-Cruces, Joâo Martins, Jorge Mota, Andrea Ceciliani, Marie Murphy, Anne Vuillemin

**Affiliations:** 1 University of Extremadura, Cáceres, Spain; 2 University of Castilla la Mancha, Ciudad Real, Spain; 3 University of Cádiz, Puerto Real, Spain; 4 University of Lisbon, Lisbon, Portugal; 5 University of Porto, Porto, Portugal; 6 University of Bologna, Bologna, Italy; 7 Ulster University, Belfast, United Kingdom; 8 Université Côte d'Azur, LAMHESS, France

**Keywords:** physical activity, sedentarism, diet, sleep, childhood, adolescence

## Abstract

Physical inactivity is a worldwide public challenge and a leading risk factor for overweight and obesity. Despite the well-recognized benefits of physical activity (PA), only 29% of European youth meet recommended guidelines of at least 60 minutes of daily moderate-to-vigorous PA. The school setting provides an ideal environment to promote healthy lifestyles among young people as initiatives can target all students and the whole school community. The main goal of the EUMOVE project is to design and implement a comprehensive set of strategies and resources to enable the educational community to promote healthy lifestyles in order to reduce risk factors for non-communicable diseases.

EUMOVE project (https://eumoveproject.eu/) is a 3-year project delivered by a collaboration between academic and non-governmental institutions from Spain, Portugal, France, Italy, and the United Kingdom. The direct beneficiaries of the project will include school leaders, teachers, and parents, and indirect beneficiaries will be primary and secondary students. EUMOVE project will develop a set of strategies and resources such as Physically Active Lessons Toolkit, Real Time Active Breaks Platform, Active School Commuting Toolkit, Learning Units about healthy lifestyles promotion, School Leaders toolkit, Parents Toolkit about promoting healthy lifestyles, and Mobile phone APP. Project dissemination will be implemented across each partner region and includes: Online dissemination through a learning platform, scientific events aimed at teachers and researchers, workshops with teachers including training on how to use the teaching resources, and Workshops with parents providing recommendations for promoting healthy lifestyle in their children.

Thus, the EUMOVE project offers evidence-based and innovative resources to be applied by the educational community in the real-world setting to promote PA levels, appropriate diet and sleep habits, and reduce sedentary time amongst children and adolescents across a number of European countries.

